# Bringing Home Baby Euclid: Testing Infants’ Basic Shape Discrimination Online

**DOI:** 10.3389/fpsyg.2021.734592

**Published:** 2021-12-20

**Authors:** Agata Bochynska, Moira R. Dillon

**Affiliations:** Department of Psychology, New York University, New York City, NY, United States

**Keywords:** change detection, geometry, online study, shape perception, infants

## Abstract

Online developmental psychology studies are still in their infancy, but their role is newly urgent in the light of the COVID-19 pandemic and the suspension of in-person research. Are online studies with infants a suitable stand-in for laboratory-based studies? Across two unmonitored online experiments using a change-detection looking-time paradigm with 96 7-month-old infants, we found that infants did not exhibit measurable sensitivities to the basic shape information that distinguishes between 2D geometric forms, as had been observed in previous laboratory experiments. Moreover, while infants were distracted in our online experiments, such distraction was nevertheless not a reliable predictor of their ability to discriminate shape information. Our findings suggest that the change-detection paradigm may not elicit infants’ shape discrimination abilities when stimuli are presented on small, personal computer screens because infants may not perceive two discrete events with only one event displaying uniquely changing information that draws their attention. Some developmental paradigms used with infants, even those that seem well-suited to the constraints and goals of online data collection, may thus not yield results consistent with the laboratory results that rely on highly controlled settings and specialized equipment, such as large screens. As developmental researchers continue to adapt laboratory-based methods to online contexts, testing those methods online is a necessary first step in creating robust tools and expanding the space of inquiry for developmental science conducted online.

## Introduction

Online studies with adults have been around in psychological research for many years, and many web-based solutions have been validated for adult testing (Buhrmester et al., 2011; Crump et al., 2013; de Leeuw et al., 2014; Gureckis et al., 2016; Sauter et al., 2020). Online studies with infants and children, however, are a relatively recent development that became newly urgent in the light of the COVID-19 pandemic and the suspension of in-person research (Lourenco and Tasimi, 2020; Sheskin et al., 2020; Zaadnoordijk et al., 2021). Because infants and young children cannot simply read the instructions and click through web-based tasks unsupervised, different solutions have been proposed for collecting developmental data online. For example, commercial or custom-built video-chat software allows an experimenter to interact with a participant through a webcam in real time while running a study remotely (Sheskin and Keil, 2018). Online platforms for unmoderated developmental research (Scott et al., 2017; Scott and Schulz, 2017; Rhodes et al., 2020; Lo et al., 2021); moreover, present detailed instructions addressed to parents or guardians allowing them to participate with their children from their home computer with a webcam, without the experimenter present and without an appointment. Several questions naturally arise: Is there a difference between online and in-laboratory results? Are there comparative advantages or unique limitations to either context? Indeed, can we ask new questions now that the space of inquiry has expanded?

Several recent online studies have found results that are mostly consistent with in-laboratory results using either the moderated video-chat or the unmoderated approach. These studies have nevertheless adapted forced-choice paradigms with children or looking-time paradigms with older infants and toddlers like preferential or “violation-of-expectation” paradigms (Scott et al., 2017; Sheskin and Keil, 2018; Leshin et al., 2020; Nussenbaum et al., 2020; Lo et al., 2021; Smith-Flores et al., 2021). Such results thus do not address whether other common methods used in developmental research, for example, some looking-time paradigms with younger infants, may be adaptable to online contexts and serve as a replacement for in-person, laboratory testing. In the present study, we thus ask whether certain early emerging abilities to discriminate shape information, which is foundational both for infants’ everyday interactions with objects (e.g., Quinn and Eimas, 1997; Quinn et al., 2001; Smith, 2009) as well as for children’s later achievement in STEM (Science, Technology, Engineering, and Mathematics) fields (e.g., Verdine et al., 2017), and which have been revealed through highly controlled laboratory studies with specialized setups and equipment, might also be measurable using unmonitored online testing, relying only on a personal computer with a webcam.

To address this question, we adapted two experiments with 7-month-old infants from a series of experiments on infant shape discrimination conducted in a laboratory setting (Dillon et al., 2020). These experiments used a “change-detection” looking-time paradigm (after Ross-Sheehy et al., 2003), in which rapidly changing displays presented visual forms (triangles or open “V” figures) on a large projector screen. On one side of the screen, the visual forms were changing in shape and area, while on the other side of the screen, the visual forms were changing in area only. On both sides, the figures were additionally changing in position and orientation. The rationale behind this paradigm is that if infants look longer at the stream of figures with the one additional change (in this case, the shape change), then that serves as evidence of their detection of that change. Dillon et al. (2020) observed across four experiments that infants showed significantly more looking to the figure streams with a shape-and-area change compared to an area-only change in full triangles and in “V” figures with relative length changes.

The change-detection paradigm has been used to investigate a variety of infants’ abilities in laboratory settings, including their sensitivity to mirror reversals in visual forms (Lauer et al., 2015), to numerical differences in dot arrays (Libertus and Brannon, 2010; Schröder et al., 2020), and to bound color and object information in visual short-term memory (Ross-Sheehy et al., 2003). It has also been used in the laboratory to chart developmental changes in infancy, e.g., in numerical discrimination from 6 to 9 months (Libertus and Brannon, 2010) and in visual short-term memory from 4 to 13 months (Ross-Sheehy et al., 2003). Moreover, small-scale longitudinal studies in the laboratory have relied on change detection to measure infants’ individual sensitivities to number and geometry, and these studies have revealed stable change detection across individuals in infancy and correlations between change detection in infancy and performance on standardized measures of symbolic mathematics in young childhood (Starr et al., 2013; Lauer and Lourenco, 2016). With the possibility that online testing will allow for larger sample sizes and the ability to collect repeated measures with the same infants over longer periods of time compared to in-laboratory testing (Sheskin et al., 2020), change detection thus becomes a prime candidate for supporting large-scale, longitudinal studies focusing on development and individual differences across domains.

The change-detection paradigm, moreover, offers additional scientific and practical advantages relative to other looking-time paradigms used with infants, like the “habituation” paradigm, which has also been used extensively in the laboratory to measure infants’ numerical and spatial sensitivities. For example, studies using habituation to evaluate infants’ shape discrimination (Schwartz and Day, 1979; Cohen and Younger, 1984; Slater et al., 1991) have relied on long presentations times, considerably longer than those reflected in natural viewing (Yu and Smith, 2016). The rapid displays used in change detection, in contrast, better reflect the dynamically changing visual world of infants’ everyday life. Moreover, the change-detection paradigm may result in lower numbers of excluded participants (Lauer et al., 2015), permits the use of other measurement tools such as automated eye tracking, and relies on fixed-duration presentations, which allow for offline coding, fewer research personnel, and even remote, unmonitored data collection.

It nevertheless remains an open question whether change detection can be adopted for online testing. In particular, most studies using change detection in the laboratory have relied on stimuli being presented on two separate monitors (e.g., Ross-Sheehy et al., 2003; Libertus and Brannon, 2010) or on a very large projector screen (e.g., Lauer et al., 2015; Dillon et al., 2020), neither of which are typically present in the home. Those that have relied on smaller screens (e.g., Schröder et al., 2020) have failed to find some of the same change-detection capacities that were found with larger screens, and unpublished data suggest that change-detection findings in the numerical domain measured in the laboratory may not robustly replicate, on either small or large screens (Lindskog et al., unpublished data). In the present study, we thus ask whether robust in-laboratory findings using change detection that presented rapidly changing, simple 2D figures on a large screen could be found using unmonitored, online data collection with stimuli presented at home on small, personal computer screens.

The present study includes two sequential experiments, one modeled after Experiment 1B and one modeled after 2B from Dillon et al. (2020). Both of these experiments produced robust findings in the laboratory; they were replications and extensions of Experiment 1A and Experiment 2A also from Dillon et al. (2020). All four of these experiments, moreover, yielded similar and medium-to-large effect sizes (Cohen’s *d*s: Experiment 1A: 0.71; Experiment 1B: 0.66; Experiment 2A: 0.68; Experiment 2B: 0.98). The methods and analysis plans for both of the present experiments were preregistered on the Open Science Framework prior to data collection,^1^ and the data and analysis code are publicly available (data: https://osf.io/ecyfd/; analysis code: https://osf.io/munk7/). The first experiment was conducted on the unmonitored online developmental testing platform Lookit^2^ when Lookit was still under development and was accessible only to a limited number of researchers. The second experiment was also conducted on Lookit, but after its beta testing had been completed and during its transition to a platform accessible to those able to comply with Lookit’s access agreement.

## General Methods

Families participated in one of two experiments through the online developmental testing platform Lookit (Scott and Schulz, 2017). They were mainly recruited by phone or email from databases of families who had expressed interest in participating in research studies, one database at Harvard University and two databases at New York University. Families were also recruited from Lookit’s participant database, posted flyers, online forums, social media sites, and word-of-mouth. They received a $5 Amazon gift card for participating. Our use of human participants was approved by the Institutional Review Boards at Massachusetts Institute of Technology (MIT; cede agreement for multi-site research at MIT and Harvard University) and at New York University. Our use of Lookit was approved initially under this cede agreement and then under Lookit’s access agreement.

**Figure 1:**
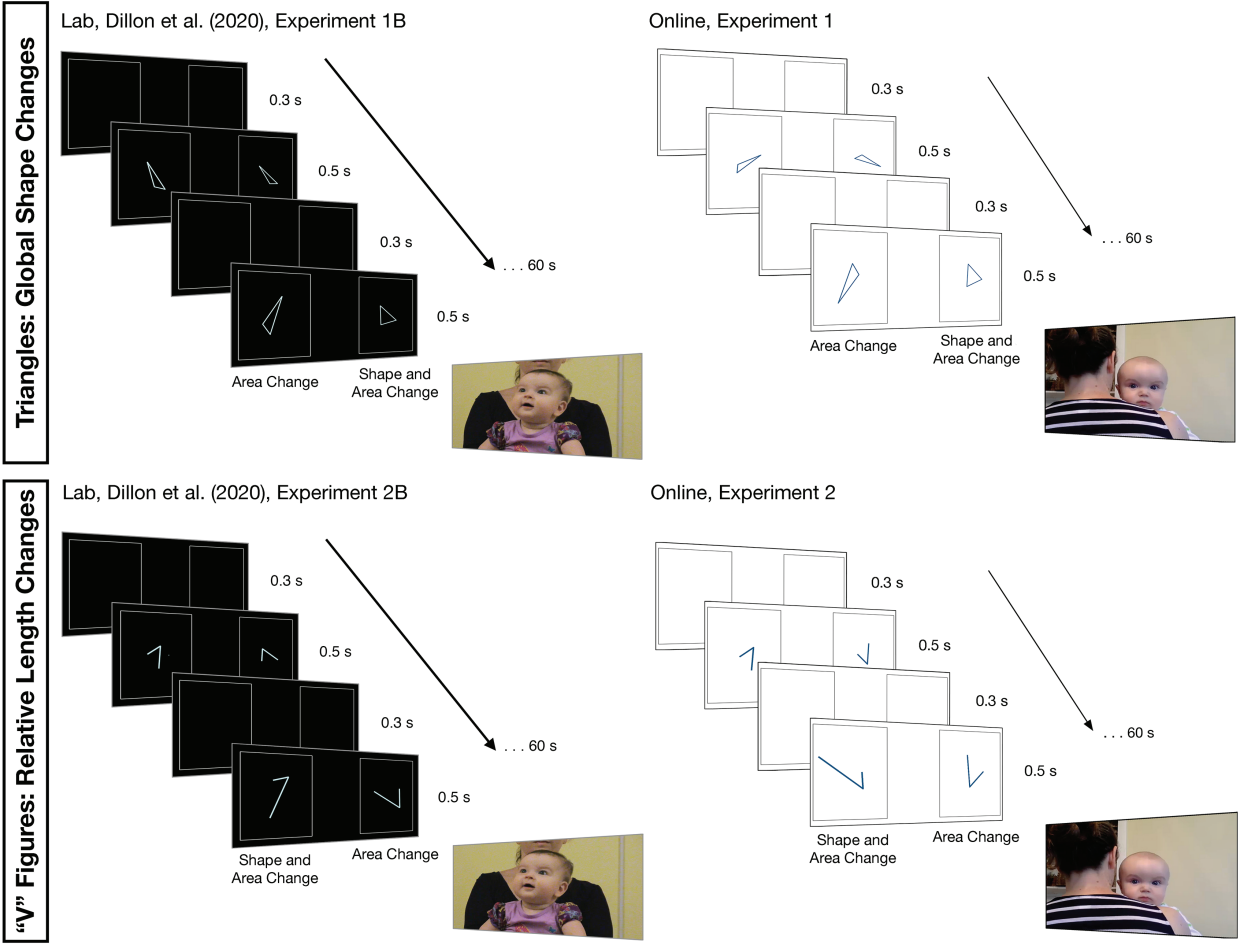
The displays and setups for the laboratory study (**left**, Dillon et al., 2020) and our present, online study (**right**) testing infants’ detection of subtle shape changes in triangles (**top**) and relative length changes “V” figures (**bottom**).

The materials and design of the experiments are illustrated in Figure 1. After Dillon et al. (2020), the experiments followed a change-detection paradigm in which dynamic streams of 2D figures appeared simultaneously, one on the left side of the screen and one on the right side of the screen, each stream bounded by a static rectangle. One stream presented figures changing in area alone (a shape-preserving scale transformation), and the other stream presented figures changing in shape and area (a shape change that resulted in an area change). For half of the infants, the area change resulted in smaller figures, and for the other half of the infants, the area change resulted in larger figures. On both sides of the screen, there was additional random variation in the figures’ positions (+/− 4.5% relative to the center of the bounding rectangle in both the vertical and horizontal directions), orientations (+/− 0–359°), and sizes (a shape-preserving scale transformation +/− 15%). While we had planned to vary figures’ left–right direction randomly for each presentation in both experiments, this variation was only implemented in Experiment 2 because of an error in Lookit’s stimuli presentation code. Each figure in each stream appeared for 0.5 s followed by a 0.3-s blank screen before the next figure appeared. Streams were presented in four 60-s blocks, and the shape change appeared on alternating sides of the screen across blocks. The shape change started on the left side of the screen for half of the infants, and it started on the right side of the screen for the other half of the infants.

Dillon et al. (2020) presented forms as light blue outlines on a black background projected on a large screen (1.07 m × 1.37 m) in a dimly lit quiet laboratory testing room. Parents sat 1.70 m from the screen, positioned infants on their laps, and closed their eyes during the stimuli presentation. They received live instruction from an experimenter who stood behind the screen and came out after each trial to reset the infant on the parent’s lap – if needed – and to recalibrate the infant’s looking. During calibration, the experimenter shook a rattle in front of different locations on the screen. Before the stimuli started, a pink circle appeared in the center of the screen, and the experimenter used the infant’s name to draw their attention to the circle (see https://osf.io/b3g52/ for example stimuli). The test trials were silent.

The differences between Dillon et al. (2020) and the present experiments are illustrated in Figure 1. In contrast to Dillon et al. (2020), our stimuli flexibly scaled to fit the screen of the personal computer on which they were being presented. To maximize visibility in the variable lighting conditions of the home-testing environment, moreover, we presented forms as dark blue outlines on a white background. Parents sat about an arm-length distance from the screen, faced away from the screen, and held their infants over their shoulders to face the screen. Our experiment, moreover, was completely unmonitored. Parents followed a set of written and pictorial descriptions instructing them how to set up the home-testing environment. Pre-recorded audio specifying the start of the experiment, the trial number, and the end of the experiment guided parents, and a twirling star with an accompanying chime sound appeared at different locations on the screen prior to each trial to calibrate infants’ looking and to introduce the test trials. Test trials were accompanied by looping infant-friendly music.

## General Preregistered Analysis

Coding and analyses followed Dillon et al. (2020). In both experiments, we measured infants’ total looking time to the figure stream presenting changes in shape and area and the stream presenting changes in area alone. Infants’ looking time to the streams was coded offline in real time from digital video recordings by a researcher masked to the changes that the infant was seeing. The total looking of 12 random infants in each experiment (25%) was recoded in their same way by a different researcher. For each infant, we calculated their proportion of looking to the shape-and-area-change stream as a function of their total looking to both streams across all four trials (see also Lauer et al., 2015). This proportion was compared to 0.50 using a one-sample, two-tailed *t*-test.

## Experiment 1

Experiment 1 adapted Experiment 1B from Dillon et al. (2020) and explored whether infants detected global shape changes in closed 2D triangles, over and above changes in triangle position, orientation, and size.

### Methods

#### Participants

Data collection took place from late March 2017 to early April 2018. Forty-eight full-term (≥37 weeks gestational age at birth) 7-month-old infants were included in the sample (22 females, mean age = 7 months 3 days, range = 6 months 15 days to 7 months 15 days). The planned sample size of 48 infants was preregistered based on a power analysis of the findings of Dillon et al. (2020), Experiment 1B (with Cohen’s *d* = 0.66 and *SD* = 0.07); power was 99.4%. For 51 families who completed the consent video, we received no test videos, and for four additional families, we received only partial test videos. Informal parental reports and discussions with the Lookit staff suggested that technical difficulties led to this large amount of missing data (due, in particular, to Lookit’s running on Adobe Flash, not HTML5, at the time). Five families completed at least one but fewer than four test blocks, and one family had poor video quality. Two additional families withdrew their consent before participating. In the corresponding laboratory study from Dillon et al., 2020, which had a sample size of 16 infants, no additional infants were excluded.

#### Displays

After Experiment 1B of Dillon et al., 2020, four triangles were used as stimuli: two similar 45°-60°-75° triangles and two similar 15°-45°-120° triangles. The areas of the smaller and larger versions of each triangle were matched across the two triangle types and differed by a factor of two (see Dillon et al., 2020, for additional details on the geometric properties of the stimuli). Each infant saw three of the four triangles. Half of the infants saw the larger 45°-60°-75° triangle on both sides of the screen, alternating with the smaller 15°-45°-120° in the shape-and-area-change stream and the smaller 45°-60°-75° triangle in the area-only-change stream. The other half of the infants saw the smaller 15°-45°-120° on both sides of the screen, alternating with the larger 45°-60°-75° triangle in the shape-and-area-change stream and the larger 15°-45°-120° triangle in the area-only-change stream.

### Results

#### Primary Preregistered Analysis

We preregistered the specification that if parents watched the test stimuli for 1 s or more, infants’ looking times would be included only up until the point at which their parent watched the stimuli for that particular block. Before analyzing our data, however, we decided to include infants’ looking times even if their parent watched the test stimuli. This more inclusive analysis, which we report in the main text, is consistent with the planned analysis, and so we report the planned analysis in the Supplementary Material.

**Figure 2:**
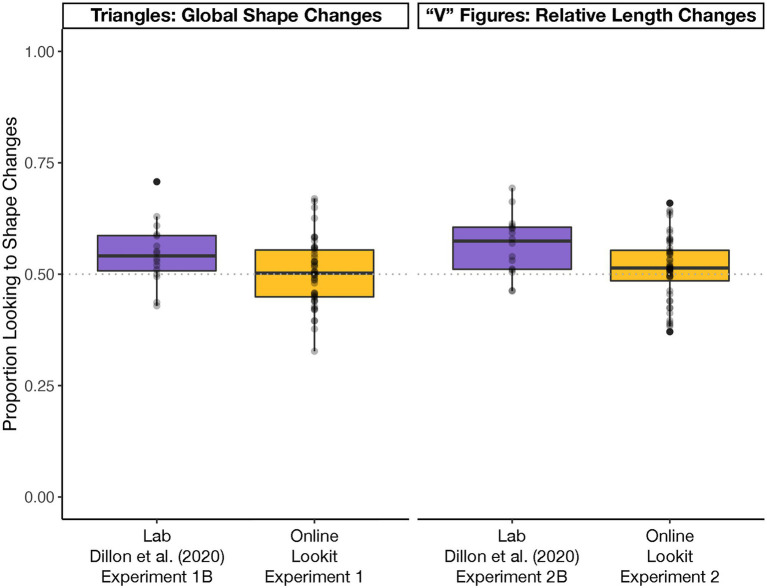
Boxplots describing the proportions of infants’ looking to shape changes (**left**) and relative length changes (**right**) in laboratory experiments, reported in Dillon et al. (2020, *N* = 16 per experiment), and in the present online experiments (*N* = 48 per experiment). They gray-dotted line at 0.50 indicates no looking preference, and the overlaid points display each participant’s individual preference, collapsed across an experiment’s four blocks. While infants looked longer at shape changes in the laboratory experiments, they did not look longer at shape changes in the online experiments.

The reliability of the two looking-time coders was high (Pearson *r* = 0.94). Unlike in Dillon et al. (2020) Experiment 1B, infants did not look significantly longer to the shape-and-area-change stream compared to the area-only-change stream [*t*(47) = 0.27, *p* = 0.786 *d* = 0.04; Figure 2A].

#### Secondary Preregistered Analysis

To further understand our findings, we first identified any influential participants by calculating Cook’s distance in a linear regression on raw total looking times to each stream for each infant with Change Type (shape-and-area change or area-only change) as a fixed effect. The analysis identified two influential participants. We reran the primary analysis after excluding these participants, and our results were consistent with the primary analysis [*t*(45) = 0.41, *p* = 0.683, *d* = 0.06].

Next, we ran a mixed-model linear regression on infants’ raw looking times after the model from Dillon et al. (2020). We had misspecified this model in our preregistration, and the correct model included Change Type (shape-and-area change or area-only change), Size (bigger triangle or smaller triangle), Block (1, 2, 3, or 4), and Gender as fixed effects, and Participant as a random-effects intercept. Consistent with the primary analysis, we found no significant effect of Change Type (*β* = 0.65, *p* = 0.503), again providing no evidence that infants looked longer to the shape-and-area-change stream compared to the area-only-change stream. There were also no significant effects of Size (*β* = 1.21, *p* = 0.363) or Gender (*β* = 1.02, *p* = 0.443), and consistent with Dillon et al. (2020), there was a significant effect of Block (*β* = −2.22, *p* < 0.001), with looking time decreasing across blocks. An additional regression using this model with incomplete datasets (we received five such datasets, but three had a condition assignment that we could not determine) showed results consistent with the primary analysis and so are reported in the Supplementary Material.

Finally, to examine whether any effects might be measurable from experiments that are shorter in duration (and thus perhaps more adaptable to online sessions) we repeated our primary analysis but only considered the first two blocks. This analysis also showed that infants did not look significantly longer to the shape-and-area-change stream compared to the area-only-change stream [*t*(47) = 0.69, *p* = 0.495, *d* = 0.10].

#### Exploratory Analysis

Our exploratory analysis specifically aimed to examine the differences between the present results and the results in Dillon et al. (2020), Experiment 1B. A direct comparison between the two experiments using an independent samples *t*-test found a significant difference between infants’ preference for the shape-and-area-change stream across the two experiments [*t*(27) = 2.10, *p* = 0.045, *d* = 0.59]. Given that our experiment differed from the original experiment in many respects as outlined above, our exploratory analyses thus focus on evaluating any effects of those differences, where possible.

First, infants looked longer at the stimuli online compared to the laboratory [*t*(33.57) = −4.78, *p* < 0.001, *d* = 1.21], suggesting that infants at least saw the stimuli for a long enough time to show the expected effect. Second, parents tested online were instructed to hold their infants over their shoulders as opposed to on their laps, and this position may have resulted in longer looking to the side of the screen away from the parent’s head, biasing the overall pattern of results. That said, about half (26/48) of parents held their child over their left shoulder for the duration of the study and three parents switched sides, so, across infants, neither side of the screen was potentially more visually accessible. Accordingly, a mixed model linear regression with Change Type (shape-and-area change or area-only change) and Side (left or right) revealed no significant effect of Side (*β* = −0.06, *p* = 0.966) and no Change Type X Side interaction (*β* = −1.22, *p* = 0.542).

Next, we focused on exploring infants’ distraction, which may have uniquely affected their ability to detect shape changes in an uncontrolled at-home environment versus a highly controlled laboratory environment. Following Scott and Schulz (2017), a researcher, masked to what infants saw and their individual looking times, recoded the videos to enumerate the following types of distracting events: fussiness (e.g., crying or squirming to get out of a parent’s lap); distracted by an external event (e.g., someone walking by); and distracted by an external object (e.g., dropping a toy or pacifier; see Scott and Schulz, 2017, for additional details). Twenty-three of the 48 infants in our sample experienced at least one distracting event (*M* = 3.61; *Median* = 2) during the experiment. A Spearman correlation revealed that the number of distracting events negatively correlated with infants’ overall looking time (*r_s_* = −0.57, *p* < 0.001). Surprisingly, a Spearman correlation also revealed that the number of distracting events negatively correlated with the proportion looking to the shape-and-area-change stream across infants (*r_s_* = −0.32, *p* = 0.025). Infants who had one or fewer distracting events (*N* = 34), moreover, showed a positive, although not significant, preference for the shape-and-area-change stream [*M* = 0.52, *SEM* = 0.01, *t*(33) = 1.73, *p* = 0.093, *d* = 0.30]. These results suggest that distraction might explain why infants in the online experiment did not show the same patterns of change detection of visual forms as were observed in the laboratory studies.

### Discussion

Unlike in Dillon et al. (2020), in Experiment 1, we found no evidence that 7-month-old infants looked significantly longer to shape changes in triangles over and above changes in triangle position, orientation, and size. To further understand how the results from Dillon et al.’s (2020) laboratory study compared to our present online study, we explored the possibility that infants were distracted in the home environments and that this distraction affected infants’ ability to detect subtle shape changes in rapidly presented displays of 2D figures. We found that the number of times that infants were distracted during the stimuli presentation negatively correlated with their ability to detect shape changes.

In Experiment 2, we thus focused on two aims. First, we focused on an experiment from Dillon et al. (2020) that probed infants’ detection of relative length changes instead of global shape changes. Relative length changes are also robustly detected in the laboratory, they can be instantiated in simpler 2D figures, and they may underlie infants’ detection of global shape changes in triangles (Dillon et al., 2020). Second, inspired by the exploratory analysis of Experiment 1, we focused on distraction specifically as the cause of the difference between the findings of the in-laboratory versus online versions of the experiment. We did so by improving the instructions given to the parents to minimize possible distractions in the home, and we introduced new exclusion criterion based on distraction, with planned analyses that allowed us evaluate the effects of distraction directly.

## Experiment 2

Experiment 2 adapted Experiment 2B from Dillon et al. (2020) and explored whether infants detected shape changes, instantiated as changes to the relative lengths of the arms forming open 2D “V” figures, over and above changes in figure position, orientation, sense, and size. As a result of the methods and exploratory findings of Experiment 1, Experiment 2 also included improved instructions to parents and preregistered exclusion criteria and analyses based on infant distraction and potential parent interference.

### Methods

#### Participants

Data collection took place from late March 2020 to late November 2020. Because of the null result in Experiment 1 and how resource intensive data collection is with infants, we preregistered a sequential sampling procedure. We used a Bayes Factor Design Analysis for sequential designs (see Stefan et al., 2019) using *r* = √2/2 as a default prior distribution on effect size *δ*, which we estimated as 0.35. After every eight infants who met the inclusion criteria, we evaluated a one-sample Bayesian *t*-test with a directional hypothesis, comparing infants’ proportion of looking to the shape-and-area-change stream to 0.50. We aimed for a strength of evidence of 6, which meant that we would collect data until the Bayes Factor was larger than 6 (evidence for H_1_), smaller than 1/6 (evidence for H_0_), or we reached a maximum sample size of 48 infants.

We did not meet the planned strength of evidence before reaching the maximum sample size, and so data from 48 full term (≥37 weeks gestational age at birth) 7-month-old infants were included in the sample (24 females, mean age = 6 months 28 days, range = 6 months 15 days to 7 months 15 days).

For 12 families who completed the consent video, we received no test videos, and for 34 additional families, we received only partial test videos. Discussions with the Lookit staff suggested that technical difficulties led to this large amount of missing data. Three families completed at least one but fewer than four blocks, and three families had poor video quality. In addition, a large number of infants (42) were excluded based on the new preregistered exclusion criteria motivated by the exploratory analysis of the effects of infant distraction from Experiment 1: eight infants with looking times <80 s; seven infants with parents who watched the test stimuli; nine infants who were distracted; two infants with looking times <80 s who were distracted; one infant with looking time < 80 s whose parents watched the test stimuli; two infants with looking times <80 s who were distracted and whose parents watched the test stimuli; and 11 infants who were distracted and had parents who watched the test stimuli. In the corresponding laboratory study from Dillon et al., 2020 (*N* = 16) one additional infant was excluded because of low looking time, one because of a preference score of more than two standard deviations above or below the mean, and one because of equipment failure.

#### Displays

After Dillon et al., 2020, Experiment 2B, four “V” figures were used, all with an angle measure of 53.39° (see Figure 1). Two of those figures had an arm-length ratio of 1:1.5 and two had an arm-length ratio of 1:3, so the relative length difference between the two figure types was 1:2. For each of the two figure types, there was one version that has a smaller implied area (formed by joining the endpoints at the open side of the “V” to make a triangle) and one that has a larger implied area (see Dillon et al., 2020, for additional details on the geometric properties of the stimuli). Each infant saw three of the four “V” figures. Half of the infants saw the larger 1:3 “V” figure on both sides of the screen, alternating with the smaller 1:1.5 “V” figure in the shape-and-area-change stream and the smaller 1:3 “V” figure in the area-only-change stream. The other half of the infants saw the smaller 1:1.5 “V” figure on both sides of the screen, alternating with the larger 1:3 “V” figure in the shape-and-area-change stream and the larger 1.1.5 “V” figure in the area-only-change stream.

### Results

#### Primary Preregistered Analysis

The reliability of the two looking-time coders was high (Pearson *r* = 0.96). Unlike in Dillon et al. (2020), infants did not look significantly longer to the relative length-and-area-change stream compared to the area-only-change stream [*t*(47) = 1.29, *p* = 0.205, *d* = 0.19; BF = 0.339; Figure 2B].

#### Secondary Preregistered Analyses

As in Experiment 1, we first identified influential participants by calculating Cook’s distance in a linear regression on infants’ raw looking times to each stream with Change Type (relative length-and-area change or area-only change) as a fixed effect. The analysis identified five influential participants. We reran the primary analysis on the data after removing these influential participants, and our results were consistent with the primary analysis [*t*(42) = 0.71, *p* = 0.481, *d* = 0.11].

We next ran a mixed-model linear regression on infants’ raw looking times with Change Type (relative length-and-area change or area-only change), Size (bigger “V” or smaller “V”), Block (1, 2, 3, or 4), and Gender as fixed effects, and Participant as a random-effects intercept. Consistent with the primary analysis, we found no significant effect of Change Type (*β* = 0.88, *p* = 0.377), indicating that infants did not look longer to the relative length-and-area-change stream compared to the area-only-change stream. There was no significant effect of Size (*β* = 0.69, *p* = 0.557) or Gender (*β* = 1.86, *p* = 0.117), but there was a significant effect of Block (*β* = −2.27, *p* < 0.001), with looking time decreasing across blocks. As in Experiment 1, we also conducted this regression including partial datasets from infants in the planned age range (we received three such datasets), and since these results were consistent with the primary analysis, they are reported in the Supplementary Material. Finally, we ran a mixed-model linear regression with the same variables in the Bayesian framework. It revealed results consistent with the hypothesis-testing framework, with an estimate of 0.88 s (95% CI: −1.07 – 2.83) for the effect of Change Type, an estimate of 0.69 s (95% CI: −1.56 – 2.94) for the effect of Size, an estimate of 1.86 s (95% CI: −0.39 – 4.11) for the effect of Gender, and an estimate of −2.27 s (95% CI: −3.15 – −1.40) for the effect of Block on infants’ looking times. As in Experiment 1, moreover, we repeated the primary analysis considering only the first two blocks. This analysis also showed that infants did not look significantly longer to the relative length-and-area-change stream compared to the area-only-change stream [*t*(47) = 0.82, *p* = 0.415, *d* = 0.19].

Finally, to evaluate the effects of distraction on infants’ performance, we repeated Experiment 2’s primary analysis but this time included the “distracted” infants, who would have met the inclusion criteria from Experiment 1. As outlined above, this sample included an additional 42 infants, and with this larger group of infants (*N* = 90) we still did not find evidence that infants looked significantly longer to the relative length-and-area-change stream compared to the area-only-change stream [*t*(89) = 1.88, *p* = 0.063, *d* = 0.20; BF = 0.630]. To examine whether the findings of our exploratory analysis of distraction from Experiment 1 generalized to Experiment 2, we used a Spearman correlation predicting looking times by the number of distracting events, as in Experiment 1, with the expanded sample of 90 participants. There was no correlation between the number of distracting events and infants’ preference for the relative length-and-area-change stream (*r_s_* = −0.09, *p* = 0.402; BF: 0.300).

#### Exploratory Analysis

To complement the exploratory analyses from Experiment 1, we first directly compared the results from this experiment to those of Dillon et al. (2020), Experiment 2B, using an independent samples *t*-test. While the difference between the two experiments was not significant [*t*(21) = 1.97, *p* = 0.062, *d* = 0.64], the effect size was medium-to-large and similar (indeed slightly larger) than the effect size characterizing the difference between Experiment 1 to Experiment 1B of Dillon et al. (2020), which did show a significant difference. As in Experiment 1, infants looked longer at the stimuli online compared to the laboratory [*t*(47.82) = −9.78, *p* < 0.001, *d* = 2.14], suggesting that they saw the stimuli for a long enough time to show the expected effect.

We next evaluated whether infants looked longer to one side of the screen and whether the number of distracting events led to differences in overall looking, not just longer looking to the relative length-and-area-change stream. A little over half of parents (29/48) held their child over their left shoulder, and a mixed-model linear regression with Change Type (relative length-and-area change or area-only change) and Side (left or right) revealed a significant effect of Side (*β* = 3.76, *p* = 0.009), with infants looking more to the right versus left side of the screen. Nevertheless, there was no Change Type X Side interaction (*β* = 0.20, *p* = 0.921), suggesting that infants did not look significantly longer at the right side of the screen, for example, when that side presented relative length-and-area changes versus area-only changes, consistent with our primary finding. Finally, while the number of distracting events did not negatively correlate with a preference for the relative length-and-area-change stream, it did positively correlate with infants’ total looking time (*r_s_* = −0.34, *p* = 0.001).

## Discussion

Unlike in Dillon et al. (2020), in Experiment 2, we found no evidence that 7-month-old infants looked significantly longer to shape changes instantiated as changes in the relative lengths of the arms forming simple 2D “V” figures. These results are consistent with Experiment 1, which also failed to find that infants could detect subtle shape changes in 2D closed figures when tested online in their home environment. Experiment 2’s null finding emerged regardless of its strict criteria excluding a large number of infants who experienced more than one distracting event during the testing session. Unlike Experiment 1, moreover, we found no relation between the number of times that infants were distracted and their ability to detect shape changes. This finding suggests that other factors, instead of or in addition to distraction, may affect infants’ performance in home versus laboratory settings.

## General Discussion

Two experiments on young infants’ shape discrimination adapted for an unmonitored online testing platform did not reveal infants’ sensitivities to shape information as had been revealed robustly in laboratory experiments. In particular, unlike in Dillon et al. (2020), we found no evidence that 7-month-old infants looked significantly longer to the shape changes in triangles (Experiment 1) or the relative length changes in “V” figures (Experiment 2) over and above changes in figure position, orientation, and size. While exploratory analyses in Experiment 1 suggested that infants’ failure to detect these shape changes might be due to their distraction, planned analyses in Experiment 2 found no relation between infant distraction and their change detection. Our findings suggest that other factors, instead of or in addition to distraction, may have instead affected infants’ performance when tested online.

One possible factor that may have limited infants’ success is the stimuli’s presentation on small, personal computer screens. For example, while Smith-Flores et al. (2021) found looking-time results with toddlers tested online that were largely consistent with laboratory-based results, they speculated that their one null-finding – that infants failed to look longer at events in which an object appeared to move through another object after rolling down a ramp – may have been due to the events’ being presented on a small screen, which minimized the visibility and salience of the violating object’s trajectory. Similarly, the small screens used in the present study may have limited the visual saliency of the subtle shape changes. Indeed, the use of small screens in such cases may affect infants’ performance whether or not they are tested online. Follow-up studies presenting different kinds and magnitudes of spatial information conducted in the laboratory using small screens may begin to address this possibility.

Among other developmental paradigms using looking time, moreover, change detection, in particular, relies on conveying that there are two discrete events being presented, with only one event displaying uniquely changing information that would draw infants’ attention. Small screens may make this important aspect of the change-detection paradigm more difficult to convey, especially compared to contexts in which change-detection displays are presented on specialized equipment, like large projector screens or two separate monitors, as had been done in most laboratory studies. While the change-detection paradigm may have seemed ideal for adaptation to online testing, in particular, because of its ability to yield reliable individual differences in longitudinal studies and its use of fixed duration trials, it may not be adaptable to online contexts or even other laboratory or field contexts if the testing in those contexts relies on small screens. Future laboratory studies presenting the same display used in Dillon et al. (2020) but on small screens may further clarify the role of screen size in eliciting infants’ change detection of shape information.

Some developmental paradigms used with young infants, even those that seem well-suited to the constraints and goals of online data collection, may thus not yield results consistent with laboratory results that rely on highly controlled settings and specialized equipment, such as large screens. Testing those paradigms online is a necessary first step in creating robust tools and expanding the space of inquiry for developmental science conducted online. As the present study suggests, moreover, such investigations may also suggest limits to developmental paradigms that are not specific to online testing but have not yet been recognized in the laboratory. Such findings thus allow us to further refine both sets of tools and better understand the contexts in which infants’ abilities can be reliably and robustly measured.

## Data Availability Statement

Example stimuli, preregistrations, data, and analysis code are publicly available at: https://osf.io/vvaw7/.

## Ethics Statement

Our use of human participants was approved by the Institutional Review Boards at Massachusetts Institute of Technology (MIT; cede agreement for multi-site research at MIT and Harvard University) and at New York University. Informed consent was obtained from parents or legal guardians for infants’ participation in this study as well as for the publication of any potentially identifiable images.

## Author Contributions

Both authors designed the study, implemented the study, analyzed the data, and wrote the paper.

## Funding

This work was supported by a National Science Foundation CAREER Award (DRL-1845924; to MRD) and a Jacobs Foundation Early Career Fellowship (to MRD).

## Conflict of Interest

The authors declare that the research was conducted in the absence of any commercial or financial relationships that could be construed as a potential conflict of interest.

## Publisher’s Note

All claims expressed in this article are solely those of the authors and do not necessarily represent those of their affiliated organizations, or those of the publisher, the editors and the reviewers. Any product that may be evaluated in this article, or claim that may be made by its manufacturer, is not guaranteed or endorsed by the publisher.

## Footnotes

^1^ https://osf.io/vvaw7/registrations

^2^ https://lookit.mit.edu/
