# Supplementary material for: Bringing Home Baby Euclid: Testing Infants’ Basic Shape Discrimination Online
Source: Front Psychol. 2021 Dec 20;12:734592. doi: 10.3389/fpsyg.2021.734592 (PMC8734637; doi:10.3389/fpsyg.2021.734592)
Supplement: Supplementary file 1 [file Data_Sheet_1.docx]

**Supplementary Materials**

**Bringing Home Baby Euclid: Testing Infants’ Basic Shape Discrimination Online**

Agata Bochynska and Moira R. Dillon

**Experiment 1**

In Experiment 1, we preregistered an analysis in which an infant’s looking time within a block was considered only up until the point at which their parent watched the test stimuli for 1 s or more. Eleven out of the 48 infants had a parent watch the test stimuli for 1 s or more, and when their subsequent looking times were removed, our results remained consistent with the results reported in the main text. We found no evidence of infants’ looking longer to the shape-and-area-change stream compared to the area-only change stream (*t*(47) = 0.50, *p* = 0.620, *d* = 0.07).

Because the study was accessible to anyone with a Lookit account, there was the possibility that additional participants who did not meet our preregistered inclusion criteria could also submit data. Only one infant in the age range who did not meet our initial inclusion criteria submitted data (an infant born before 37 weeks gestational age). Moreover, only two infants outside of the preregistered age range submitted data. So, we decided not to run the additional planned analyses including these data.

We planned to run a regression on raw looking times with partially completed datasets in addition to our main regression, which only included complete datasets. We received five partial datasets, three whose condition assignment could not be determined. Consistent with the primary analysis, the analysis with the two additional partial datasets revealed no significant effect of Change Type (*β* = 0.41, *p* = .670), Size (*β* = 1.14, *p* = .375), or Gender (*β* = 0.90, *p* = .483), but a significant effect of Block (*β* = -2.22, *p* < .001), with looking time decreasing across blocks.

We preregistered an analysis to evaluate test-retest reliability of infants’ shape-change detection if a significant number of participants completed the experiment more than once. No participants completed the experiment more than once so we did not conduct this analysis.

**Experiment 2**

We planned to run a regression on raw looking times with partially completed datasets in addition to our main regression, which only included complete datasets. We received three partial datasets. Consistent with the primary analysis, the analysis with the three additional partial datasets revealed no significant effect of Change Type (*β* = 1.09, *p* = .275), Size (*β* = 0.79, *p* = .493), or Gender (*β* = 1.81, *p* = .121), but a significant effect of Block (*β* = -2.30, *p* < .001), with looking time decreasing across blocks. The Bayesian framework revealed results consistent with the hypothesis-testing framework, with an estimate of 1.09 s (95% CI: -0.86 – 3.04) for the effect of Change Type, an estimate of 0.80 s (95% CI: -1.43 – 3.01) for the effect of Size, an estimate of 1.80 s (95% CI: -0.41 – 4.03) for Gender, and an estimate of -2.30 s (95% CI: -3.17 – -1.43) for the effect of Block on infants’ looking times.

We preregistered an analysis to evaluate test-retest reliability of infants’ shape-change detection if a significant number of participants completed the experiment more than once. Only one participant completed the experiment more than once so we did not conduct this analysis.
